# Factors Associated with High Sodium Intake Assessed from 24-hour Urinary Excretion and the Potential Effect of Energy Intake

**DOI:** 10.1155/2019/6781597

**Published:** 2019-05-02

**Authors:** Fatimah Othman, Rashidah Ambak, Cheong Siew Man, Nor Azian Mohd Zaki, Mohd Hasnan Ahmad, Nur Shahida Abdul Aziz, Azli Baharuddin, Ruhaya Salleh, Tahir Aris

**Affiliations:** Centre for Nutrition Epidemiology Research, Institute for Public Health, Ministry of Health Malaysia, Kuala Lumpur, Malaysia

## Abstract

Dietary consumption and other environmental factors are known factors associated with sodium intake. However, little is known about the influence of energy intake on this relationship. The aim of this study was to determine the risk factors associated with high sodium intake assessed from urine sodium excretion and the influence of energy intake. A nationwide, cross-sectional study was conducted from 2015 to 2016 among Malaysian health staff (MySalt 2015). A total of 1027 participants from 1568 targeted participants aged 18 years and older that were randomly selected were included in this study. Sodium intake was determined by measuring sodium excretion in the 24 hr urine test. Dietary, sociodemography, and anthropometry variables as associated risk factors were assessed. Multiple logistic regression models were used to determine the association between high sodium intake (≥2000 mg/day urinary sodium) and potential risk factors. The prevalence of high sodium intake in this study was 70.1% (*n*=733). High sodium intake was associated with male (OR 1.93, 95% confidence interval (CI) 1.41, 2.64), Bumiputera Sarawak ethnicity (OR 0.24, 95% CI 0.09, 0.62), and energy-adjusted sodium intake (mg/d) (OR 1.19, 95% CI 1.03–1.39). Our results suggested that sex, ethnicity, and energy-adjusted sodium consumption were strong risk factors associated with high sodium intake independent from energy and other potential confounding factors.

## 1. Introduction

Sodium is an essential electrolyte that regulates blood volume and osmotic equilibrium in the human body. However, excessive sodium consumption can cause fluid retention and, subsequently, increased blood pressure. Conclusive scientific evidence also connects excessive consumption of sodium with cardiovascular disease, gastric cancer, osteoporosis, cataract, kidney stones, and diabetes [1].

The World Health Organization (WHO) suggests an intake of not more than 2000 mg/day of sodium to reduce blood pressure and the risk of cardiovascular disease [2]. This position has been endorsed by the United Nations High Level Meeting on Noncommunicable Diseases (NCDs) as voluntary global targets for the prevention and control of NCDs by reducing sodium intake as a core target [3].

In Malaysia, the average sodium intake among adults varies, ranging from 1935 mg/d to 3429 mg/d [4, 5]. Based on these findings, sodium intake among Malaysian adults is far lower than the average sodium intake in other Asian countries [6]. However, sodium intake in Malaysia generally exceeded the WHO recommendation [7]. As noncommunicable diseases (NCDs) escalate in Malaysia [8], controlling sodium intake is one of the most cost-effective ways to reduce NCD prevalence.

Twenty-four-hour urine collection is the recommended method to monitor population sodium intake. Assuming that no urine void is missed, about 90%–95% of the sodium consumed from all sources is excreted in urine and its estimation is not influenced by recall bias [9]. Sodium intake was found to be influenced by environmental factors including sociodemography, gender, and body size [10–14]. However, little is known whether the link of sodium intake was influenced by energy consumption. Recent observation suggested a higher body mass index correlated to sodium intake by stimulating the adiposity gene synthesis independent of calorie intake [14, 15]. However, conflicting hypotheses have been raised, where high sodium intake was assumed to increase thirst, which stimulates energy drink consumption leading to additional energy intake [16, 17].

Further evaluation should be explored to evaluate the potential effect of energy intake in the association of health determinants and sodium consumption, which is commonly linked to cardiovascular disease. In addition, sodium excretion as a biomarker in monitoring sodium intake is highly regulated by hormonal and renal handling, which alters sodium metabolism, absorption, and excretion [18]. For the purpose of this study, we focused the influencing factors of sociodemographic characteristic, dietary intake, and body composition status towards sodium consumption. Greater understanding of this correlation may aid the understanding of how sodium, energy intake, and other health determinants are interlinked.

## 2. Materials and Methods

### 2.1. Study Design

This cross-sectional study was conducted in 16 study sites in Malaysia, comprising 14 state health departments, Ministry of Health's headquarters in Putrajaya, and research institutes. Data were collected between November and December 2015, and the number of respondents for this study was calculated using the WHO matrix table [18]. The sample size of 1568, including the attrition rate, was estimated based on 10 mmol/day maximum difference in sodium excretion and the standard deviation of 70 mmol. Based on nonproportionate sampling, respondents were selected randomly from each study site. Permission to conduct this study was obtained from the Medical Research Ethics Committee (MREC), Ministry of Health, Malaysia.

### 2.2. Study Participants

The inclusion criteria of respondents were 18 years old and above, free of kidney disease, nonpregnant women, and not on diuretic therapy in the two weeks before data collection. Kidney disease was defined as having renal impairment or any chronic kidney disease diagnosed by a medical doctor prior to data collection. Written informed consent was obtained from participants, and data without personal identification were used for the analysis.

### 2.3. Measures

Each participant was visited twice. In the first visit, information on demographic characteristics (age, sex, ethnicity, income, educational attainment, and marital status) and medical history (history of diabetes, hypertension and heart, liver, and kidney diseases) was obtained by self-administered questionnaires. Ethnicity was classified based on the five biggest ethnic groups in Malaysia: Malay, Chinese, Indian, Bumiputra Sabah, and Bumiputra Sarawak. Education attainment was categorized as secondary or below, form 6 or diploma, and college or university, while marital status was classified as single and married. In the second visit, the measurement of anthropometry and blood pressures were measured, and the collected 24 hr urine was sent to the laboratory.

### 2.4. Anthropometry and Blood Pressure

Body height of the participants was measured using SECA Stadiometer 213 (Germany) to the nearest 0.1 cm. Body weight of the participants was measured to the nearest 0.1 kg using a calibrated digital electronic weighing scale (TANITA, HD 319) with light clothing, without accessories, socks, and shoes, by trained personnel. Body mass index (BMI) was calculated as weight in kilogram divided by the square of the height in meters. BMI was categorized as normal (BMI ≤ 18.5–24.99 kg/m^2^) and overweight/obese (≥BMI 25.00) [19].

Waist circumference (WC) was measured using the SECA measuring tape (SECureA 201, Germany). Abdominal obesity was classified as WC > 80 cm (women) and WC > 90 cm [20]. Blood pressure was measured using a digital automatic blood pressure monitor (Omron HEM-7221) to the nearest 0.1 units with appropriate cuff size. All measurements were done in duplicate, and mean readings were computed for data analysis.

### 2.5. Sodium Intake Measurement

Sodium intake was assessed using 24 hr urinary sodium excretion. All participants were given written and verbal instructions to collect a 24 hr urine sample. The first urine in the morning was discarded, and all urine over the following 24 hr was collected in the 2.5 L urine bottles. A complete urine sample was defined as follows: (1) a total 24 h urinary volume ≥500 mL, (2) no menstruation during the collection period, and (3) a reported length of collection >20 hours. High sodium intake was defined as urinary sodium excretion of ≥2000 mg/day and <2000 mg/day for normal sodium intake.

In the laboratory, sodium was determined using ion selective electrode diluted in Architect C System Analyzer. It has been estimated that 90–95% of sodium ingested was excreted in the urine [21]. Considering that 95% of the sodium excreted in urine, urinary sodium was multiplied by 100/95 to quantify the dietary sodium [22]. Creatinine was measured using kinetic alkaline picrate in a similar analyzer.

A two-day food diary was used to assess habitual dietary intake. For the food diary, participants were instructed to fill in their food intake during one weekend and one weekday at the same time 24 hr urine collection was completed. The participants were trained in formal study training by nutrition professionals on estimation of food amounts, portion, and details of food taken. Illustrated flip charts of local food were used as teaching aids to assist the participants to estimate their food portions intake. The two-day food diary was completed by participants and verified by the trained nutrition professionals before submission. The records were checked for discrepancies and omissions of any food item, portion size, and food brand to ensure the validity of the records.

Nutrient intake was analyzed using Nutritionist Pro TM Nutrition Analysis Software (Axxya Systems, Woodinville, WA, USA, 7^th^ version) and the Nutrient Composition of Malaysian Food Database. Reported energy intake of <500 kcal/day or >5000 kcal/day for women or >8000 kcal for men was considered under or over reporting and excluded from the analysis [23]. The analysis of dietary intake was based on the energy-adjusted residual method to avoid the confounding effect of energy intake [24].

### 2.6. Statistical Analysis

All statistical analyses were performed using the Statistical Package for Social Science SPSS version 22 (IBM Corp., Armonk, NY, USA). A normality test (Kolmogorov–Smirnov test) was applied to the continuous variables in this study. The analysis in this study was stratified by high sodium intake (≥2000 mg/day of 24 hr urine sodium) and <2000 mg/day of 24 hr urine sodium for normal intake. Baseline characteristics of study participants were compared between normal and high sodium intake using an independent *t* test for normal distributed continuous data in this study. Chi-square analysis was used to compare the frequencies for categorical variables between normal and high sodium intake.

Partial correlation was used to detect bivariate association between investigated parameters and urinary sodium by controlling the energy intake. The logistic regression test was used to identify the factors related to high sodium intake. Two different models were developed by including and excluding energy intake as covariate. Model 1 adjusted for age, sex, household income, education attainment, ethnicity, marital status, diabetes and hypertension status, body mass index, abdominal obese, systolic and diastolic blood pressure, and energy-adjusted sodium and other nutrients. To examine the effect of energy intake, model 2 additionally adjusted for energy intake in addition to all mentioned variables in model 1. The test for interaction between significant confounding factors and multicolinearity test was performed in the final analysis. All statistical tests were two-sided, and statistical significance was determined at *p* < 0.05.

## 3. Results

### 3.1. Characteristics of Participants

Of the 1568 targeted participants, 1127 participants with response rate of 71.9% were eligible and willing to take part in the study. After excluding the participants with urine collection <500 mL, a total of 1027 participants were included in the final analysis.  Table 1 shows the characteristics of participants stratified by normal and high sodium intake. There was a higher proportion of females, aged 30–39 years old, Malay ethnic, married, and without history of diabetes/hypertension, overweight/obese, and abdominal obese in this study.

**Figure 1:**
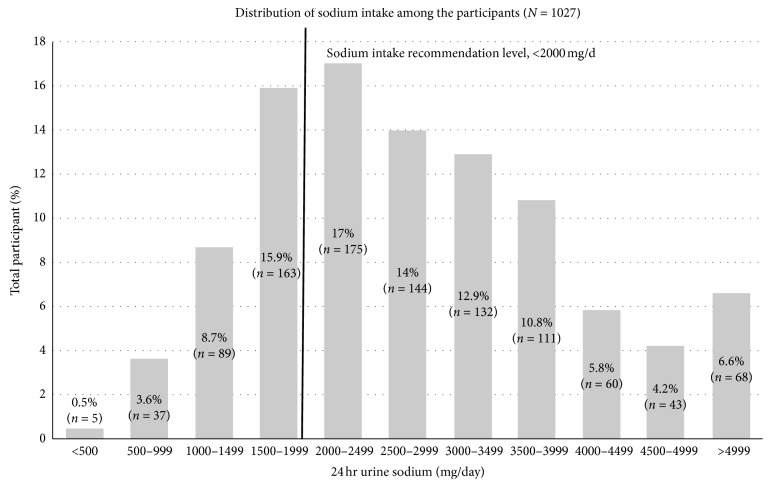
Distribution of sodium intake among the participants.

Out of 1027 participants, 29.9% of them consumed sodium lesser than 2000 mg/day. The remaining proportion or 70.1% participants consumed sodium higher than recommendation of 2000 mg/day (Figure 1).

24 hr urine sodium, creatinine, urine volume, and systolic blood pressure were significantly higher in the high sodium group compared to the normal sodium group (*p* < 0.001). The mean calorie intake, carbohydrate, protein, fat, and sodium were significantly higher in the high sodium group compared to the normal sodium group (*p* < 0.01). There was no significant difference in calcium and potassium intake between the two groups.

### 3.2. Factors Associated with High Sodium Intake

Partial correlation controlling the energy indicated that sodium consumption was significantly associated with BMI (*r* = 0.19), waist circumference (*r* = 0.22), protein (*r* = 0.08), fat (*r* = 0.08), and sodium (*r* = 0.08), and *p* < 0.05. No significant associations with carbohydrate, calcium, and potassium were found (*p* > 0.05).

Table 2 presents the associated factors related to high sodium consumption. On univariate analysis, men (OR 2.14, *p* < 0.001), Bumiputera Sarawak ethnicity (OR 0.25, *p* < 0.05), obese/overweight (BMI ≥ 25) (OR 1.45, *p* < 0.001), abdominal obese (OR 1.01, *p* < 0.05), systolic blood pressure (OR 1.20, *p* < 0.05), energy-adjusted protein (OR 1.17, *p* < 0.05), and energy-adjusted sodium intake (OR 1.17, *p* < 0.05) were more likely to have higher sodium. Age, marital status, educational level, incomes, diabetes/hypertension history, and energy-adjusted carbohydrate and potassium were not significantly associated to high sodium intake (*p* > 0.05).

Multivariate logistic regression models in Table 3 summarise the association of investigated factors and high sodium intake by including and excluding energy intake as covariate in the model. Men (OR 1.93, *p* < 0.001), Bumiputera Sarawak ethnicity (OR 0.24, *p* < 0.05), and energy-adjusted sodium calculated from food diary (OR 1.19, *p* < 0.05) remained robust and independent of the energy consumption for the high sodium intake in the final model. The assumption of model was met, and no significant interaction and multicolinearity were shown.

## 4. Discussion

A current study showed 70.1% of the participants had high sodium intake. The mean of urinary sodium was 2846 mg/day, equivalent to 7150 mg salt, exceeding the WHO-recommended sodium intake of 2000 mg/day [25]. This finding, however, was lower than the previous study conducted among normotensive working adults in the Ministry of Health, Malaysia, in 2012, which was 3430 mg/day [5] and population intake in other countries, such as China, Japan, United Kingdom, Northern Ireland, and USA, in the INTERMAP study [26].

Men had higher sodium intake than women. Previous studies also found men consumed higher quantities of sodium than women [27, 28]. In this study, men showed 2.14 times higher risk than women to have higher sodium consumption and remained significant after adjusting all potential confounding factors in the final model.

High sodium intake among men might possibly be due to higher energy and sodium intake; however, controlling energy consumption in the final model did not alter the association between gender and high sodium intake. There was also no significant interaction between gender and dietary intake. The consistent findings suggested the influence of renal sodium reabsorption and water retention that need to be considered when interpreting sodium intake assessed from urine.

In the final model of analysis, the Bumiputera Sarawak ethnic group showed lower risk (OR 0.24, *p* < 0.05) for high sodium intake compared to other ethnicities. Lowest sodium intake assessed from food diary (1966 ± 845 mg/day, *p* > 0.05) in Bumiputera Sarawak compared to the other ethnicities might be possible factors explaining this observation.

Age, education attainment, and income were not significantly associated with high sodium intake. Increase in age was connected to high sodium consumption as older adults tend to lose sense of taste and smell, hence increasing their preference of salty-tasting food [29]. Inversely, the lowest prevalence of high sodium excretion in this study was seen among participants aged 50–59 years old, compared to the younger age category (*p* > 0.05).

Our study did not find a significant association between education attainment and high sodium intake, as opposed to previous studies that related socioeconomic status (SES) with health inequalities, salt consumption, and hypertension [30, 31]. This could be due to difficulties in gaining information on health risk prevention among population with low SES [32].

In this study, a significant difference of 24 hr urine sodium between single (2650.38 ± 1323 mg/day) and married status (2914.12 ± 1378 mg/day) was observed (*p* < 0.05). It was expected that married individuals cooked a greater variety of dishes at home and thus took more salt. However, marital status was not significantly associated with high sodium consumption. Similar findings were observed in other studies [33, 34].

Excessive sodium consumption can increase blood pressure [35, 36]. However, the final model analysis showed systolic and diastolic measurements were not significantly associated with high sodium consumption. The blood pressure responses to sodium intake changes are heterogeneous and influenced by a different phenotype of salt sensitivity [37].

Univariate analysis in this study demonstrated a significant association between energy-adjusted sodium and protein with high sodium consumption. Energy-adjusted dietary sodium intake remained significantly associated with high sodium status even after including energy intake in the final model. It suggested the independent effect of sodium from the energy intake to higher sodium consumption in this study.

Sodium intake was connected to obesity due to the amount of food consumption that contains more calories and sugar^.^ [16, 17]. A study in UK reported one g/day increase of salt intake had been observed to elevate obesity risk by 26% and 0.91 kg body fat mass in adults after adjusting the possible confounding factors including energy intake [38]. Current study findings showed significant association of sodium intake assessed from 24 hr urine with BMI in a partial correlation test controlling energy intake. Univariate analysis also indicated an increased risk for high sodium among obese/overweight and abdominal obese. However, neither obesity/overweight nor abdominal obese remained as significant factors for high sodium intake after including energy intake in the final multivariate analysis. This finding supports the connection of obesity and high sodium intake possibly mediated by energy intake as observed in other studies [16, 17].

There are limitations that need to be considered when interpreting our results. The sodium intake in this study was based on an urban living homogenous population with an almost identical lifestyle, dietary practice, and medical history. Therefore, the results cannot be generalized to the general population. Although the test in this study used recommended 24 hr urine sodium excretion for sodium intake assessment, it imposed an inaccurate urine collection, such as missing urine, and was taken in single collection, which could not represent a habitual individual sodium intake.

Despite these limitations, the present study has some strength. First, the dietary assessment was obtained from two days of food records to provide less-biased food intake information than a food frequency questionnaire or 24 hr diet recall. Food records also provide high-quality data without interviewer review when participants were ordered to record their food intake. In addition, the participants underwent proper training on dietary recording in this study to minimize the discrepancy of the data. We also adjusted for misreporting by excluding participants with implausible dietary intake and controlled for energy intake in each nutrient analysis using the residual method.

Second, the sample was designed to be representative of the homogenized population working in healthcare service in Malaysia, enabling the planning for sodium reduction by policy makers in this target group. Healthcare service providers are the community that could accommodate and engage with a wider range of population that seeks healthcare services. The dissemination of information to reduce sodium intake among the healthcare workers will help to publicize the message to the population in a cost-effective way.

## 5. Conclusions

We identified an independent association of male sex, ethnicity (Bumiputera Sarawak), and energy-adjusted sodium with high sodium intake in urban, homogenous subpopulation of Malaysian adults. The attenuation of obesity as a risk factor in high sodium intake after adjusting the energy consumption suggested the significant role of energy intake when investigating sodium and obesity connection.

## Figures and Tables

**Table 1:** General characteristics of the study participants stratified by sodium intake.

Parameters	All participants (*n*=1027)	Sodium <2000 mg (*n* = 294)	Sodium ≥2000 mg (*n* = 733)	*p* value
Sex, *n* (%)				
Male	406	80 (19.7%)	326 (80.3%)	<0.001^*∗∗*^

Age, *n* (%)				
20–29 years	226	65 (28.8%)	161 (71.2%)	0.754
30–39 years	451	126 (28.2%)	324 (71.8%)	
40–49 years	191	60 (31.4%)	131 (68.6%)	
50–59 years	159	42 (4.1%)	117 (11.4%)	

Ethnicity, *n* (%)				
Malay	873	240 (27.5%)	633 (72.5%)	0.023^*∗*^
Chinese	51	18 (35.3%)	33 (64.7%)	
Indian	29	8 (27.6%)	21 (72.4%)	
Bumiputera Sabah	54	16 (29.6%)	38 (70.4%)	
Bumiputera Sarawak	20	12 (60.0%)	8 (40.0%)	

Marital status, *n* (%)				
Single	206	62 (30.1%)	144 (69.9%)	0.338

Education level, *n* (%)				
Secondary and below	225	54 (24.0%)	171 (76.0%)	0.171
Form 6/diploma	413	128 (31.0%)	285 (69.0%)	
College/university	380	111 (29.2%)	269 (70.8%)	

Diabetes, *n* (%)				
Yes	47	11 (23.4%)	37 (76.6%)	0.250

Hypertension, *n* (%)				
Yes	93	21 (22.6%)	72 (77.4%)	0.520

BMI (kg/m^2^), *n* (%)				
Obese/overweight	622	152 (24.4%)	470 (75.6%)	<0.001^*∗∗*^

Abdominal obese, *n* (%)				
Yes	586	149 (25.4%)	437 (74.6%)	0.007^*∗*^

24 hr urine, mean ± SD				
Sodium (mg/d)	2860.14 ± 1369.38	1459.83 ± 386.68	3421.79 ± 1210.39	<0.001^*∗∗*^
Creatinine (mg/L)	1044.51 ± 494.59	703.30 ± 382.81	1181.36 ± 467.66	0.034
Volume (mL)	1240.49 ± 555.53	1028.16 ± 501.36	1325.65 ± 534	<0.001^*∗∗*^

Blood pressure, Mean ± SD				
Systolic BP (mmHg)	121.23 ± 17.29	119.24 ± 18.28	122.04 ± 16.83	0.019^*∗*^
Diastolic BP (mmHg)	76.94 ± 11.18	75.94 ± 11.18	77.35 ± 11.17	0.068

Dietary intake, mean ± SD				
Energy (kcal/day)	1794.31 ± 526.47	1687.28 ± 574.65	1837.35 ± 499.79	<0.001^*∗∗*^
Carbohydrate (g/d)	224.49 ± 77.87	212.26 ± 77.81	229.41 ± 77.28	<0.001^*∗∗*^
Protein (g/d)	74.88 ± 41.26	67.04 ± 32.53	78.09 ± 43.91	<0.001^*∗∗*^
Fat (g/d)	64.88 ± 24.77	61.75 ± 30.25	66.15 ± 22.09	<0.001^*∗∗*^
Sodium (mg/d)	2772.97 ± 1045.85	2561.43 ± 1065	2859.05 ± 1025.93	<0.001^*∗∗*^
Calcium (mg/d)	494.95 ± 273.26	484.39 ± 317.40	499.19 ± 253.46	0.434
Potassium (mg/d)	1182.70 ± 494.21	1141.37 ± 571.67	1199.32 ± 458.77	0.091

^*∗∗*^Significant at *p* value < 0.001. ^*∗*^Significant at *p* value < 0.05.

**Table 2:** High sodium intake predicted by sociodemography, clinical parameter, and dietary intake.

Risk factors	High sodium intake	
	OR (95% CI)	*p* value
*Sociodemographic*		
Age	1.00 (1.00, 1.02)	0.653
Sex		
Women	Reference	
Men	2.14 (1.60, 2.88)	<0.001^*∗∗*^
Marital status		
Single	Reference	
Married	0.92 (0.66, 1.28)	0.619
Ethnicity		
Malay	Reference	
Chinese	0.70 (0.38, 1.26)	0.229
Indian	0.99 (0.43, 2.28)	0.991
Bumiputera Sabah	0.90 (0.49, 1.64)	0.733
Bumiputera Sarawak	0.25 (0.10, 0.63)	0.003^*∗*^
Education level		
Secondary and below	Reference	
Form 6/diploma	0.70 (0.49, 1.02)	0.703
College/university	0.77 (0.53, 1.12)	0.765
Income	1.00	0.644
History of diabetes		
No	Reference	
Yes	1.51 (0.74, 3.09)	0.254
History of hypertension		
No	Reference	
Yes	1.17 (0.72,1.91)	0.520

*Clinical parameter*		
BMI (kg/m^2^)		
Normal	Reference	
Overweight/obese	1.69 (1.28, 2.23)	<0.001
Abdominal obese		
No	Reference	
Yes	1.45 (1.10, 1.91)	0.008^*∗*^
Blood pressure (mmHg)		
Systolic	1.01 (1.00, 1.02)	0.020^*∗*^
Diastolic blood pressure	1.01 (1.00, 1.02)	0.068
Dietary intake		
Adjusted carbohydrate (g/day)^†^	0.98 (0.86, 1.13)	0.794
Adjusted protein (g/day)^†^	1.20 (1.01, 1.44)	0.040^*∗*^
Adjusted fat (g/day)^†^	0.89 (0.77, 1.01)	0.079
Adjusted sodium (mg/day)^†^	1.17 (1.01, 1.35)	0.033^*∗*^
Adjusted potassium (mg/day)^†^	0.94 (0.82, 1.07)	0.347
Adjusted calcium (mg/day)^†^	0.94 (0.82, 1.06)	0.322

^†^Energy-adjusted value. ^*∗∗*^Significant at *p* value < 0.001. ^*∗*^Significant at *p* value < 0.05.

**Table 3:** Association between high sodium intake, sociodemography, clinical parameter, and dietary intake with and without energy intake adjustment.

Risk factors	High sodium intake			
	Model 1^†^		Model 2^††^	
	OR (95% CI)^†^	*p* value	OR (95% CI)^††^	*p* value
*Sociodemography*				
Sex				
Women	Reference		Reference	
Men	2.18 (1.60, 2.95)	<0.001^*∗∗*^	1.93 (1.41, 2.64)	<0.001^*∗∗*^
Ethnicity				
Malay	Reference		Reference	
Chinese	0.79 (0.43, 1.46)	0.456	0.85 (0.46, 1.58)	0.610
Indian	1.00 (0.42, 2.36)	0.999	1.10 (0.47, 2.60)	0.825
Bumiputera Sabah	0.87 (0.46, 1.61)	0.637	0.86 (0.47, 1.64)	0.678
Bumiputera Sarawak	0.23 (0.09, 0.57)	0.002^*∗*^	0.24 (0.09, 0.62)	0.003^*∗*^

*Clinical parameters*				
BMI (kg/m^2^)
Normal	Reference		Reference	
Overweight/obese	1.55 (1.17, 2.05)	0.003	0.73 (0.25, 2.11)	0.560
Dietary intake				
Energy-adjusted sodium (mg/day)	1.19 (1.03, 1.38)	0.019^*∗*^	1.19 (1.03, 1.39)	0.021^*∗*^

^†^Model included covariates with *p* < 0.25 in the univariate analysis. ^††^Model included energy intake and covariates with *p* < 0.25 in the univariate analysis. Model assumption was met. No multicolinearity and significant interaction were observed. ^*∗∗*^Significant at *p* value < 0.001. ^*∗*^Significant at *p* value < 0.05.

## Data Availability

The data used in this study are available from the Institute for Public Health, National Institute of Health, Malaysia, and available from the authors upon reasonable request and with permission of Director General, Ministry of Health, Malaysia.
